# The role of cannabinoid receptor 2 in bone remodeling during orthodontic tooth movement

**DOI:** 10.1186/s12903-023-03810-5

**Published:** 2024-01-04

**Authors:** Deng-ying Fan, Hao-yan Zhai, Yuan Zhao, Xing Qiao, De-chao Zhu, Hui-Juan Liu, Chunyan Liu

**Affiliations:** 1 Department of Orthodontics, School and Hospital of Stomatology, Hebei Medical University & Hebei Key Laboratory of Stomatology & Hebei Clinical Research Center for Oral Diseases, East 383 Zhongshan Road, Shijiazhuang, Hebei Province 050017 China; 2 The Key Laboratory of Stomatology, School and Hospital of Stomatology, Hebei Medical University & Hebei Key Laboratory of Stomatology, Clinical Research Center for Oral Diseases, East 383 Zhongshan Road, Shijiazhuang, Hebei Province 050017 China

**Keywords:** Cannabinoid receptors, Tooth movement, Alveolar bone remodeling

## Abstract

**Background:**

The purpose of this study is to explore the effects of CB2 on bone regulation during orthodontic tooth movement.

**Methods:**

Thirty male mice were allocated into 2 groups (*n* = 15 in each group): wild type (WT) group and CB2 knockout (CB2^−/−^) group. Orthodontic tooth movement (OTM) was induced by applying a nickel-titanium coil spring between the maxillary first molar and the central incisors. There are three subgroups within the WT groups (0, 7 and 14 days) and the CB2^−/−^ groups (0, 7 and 14 days). 0-day groups without force application. Tooth displacement, alveolar bone mass and alveolar bone volume were assessed by micro-CT on 0, 7 and 14 days, and the number of osteoclasts was quantified by tartrate-resistant acid phosphatase (TRAP) staining. Moreover, the expression levels of RANKL and OPG in the compression area were measured histomorphometrically.

**Results:**

The WT group exhibited the typical pattern of OTM, characterized by narrowed periodontal space and bone resorption on the compression area. In contrast, the accelerated tooth displacement, increased osteoclast number (*P* < 0.0001) and bone resorption on the compression area in CB2^−/−^ group. Additionally, the expression of RANKL was significantly upregulated, while OPG showed low levels in the compression area of the CB2 ^− / −^ group (*P* < 0.0001).

**Conclusions:**

CB2 modulated OTM and bone remodeling through regulating osteoclast activity and RANKL/OPG balance.

**Supplementary Information:**

The online version contains supplementary material available at 10.1186/s12903-023-03810-5.

## Introduction

Orthodontic tooth movement (OTM) is a process of correcting the position of the teeth by applying a force. It is a process involving the biomechanical adaptation of the alveolar bone remodeling and its surrounding periodontium. OTM results in remodeling of the alveolar bone through an external mechanical force, and the force induces cytokine, growth factor, and neurotransmitter release, which signal bone formation in tension areas and bone resorption in compression areas [1]. One of the key factors that regulates alveolar bone remodeling is the OPG/RANKL/RANK axis. The osteoclast differentiation was induced by RANKL binding to RANK, whereas osteoclastogenesis was inhibited by OPG binding to RANK [2–5], which are regulated by multiple factors.

CB2 is a G protein-coupled receptor that modulates the proliferation, differentiation, and maturation of osteoclasts, osteoblasts, and osteocytes [1, 6]. CB2 plays an important role in bone homeostasis and bone remodeling, which have been widely studied for the treatment of bone damage and inflammatory disorders [7, 8]. The effects of CB2 on bone remodeling are controversial, as some studies have reported that CB2 deficiency leads to decreased osteoclasts and alveolar bone density, while others have reported the opposite [9–14]. Currently the use of cannabis has become more popular, and Klein et al. (2022) reported that dronabinol, which can combine with CB2, attenuates OTM by decreasing bone resorption in rats [15]. To further explore the role of CB2 in OTM, we used *Cnr2* knockout mice in this study.

Over all, the specific research questions are as follows:

- RQ1: How does CB2 gene knockout affect orthodontic tooth movement?
- RQ2: How does alveolar bone remold under orthodontic force after CB2 gene knockout?
- RQ3: How does osteoclasts change during alveolar bone remodeling induced by orthodontic force?

## Materials and methods

This study was conducted with the ethical guidelines of the Laboratory Animal Ethical and Welfare Committee of Hebei Medical University (approval number: 20220301).

### Animals

*Cnr2* knockout mice (CB2^−/−^, C57BL/6 J background) were obtained from Gempharmatech Co., Ltd. A total of 15 male CB2^−/−^ mice (22 to 23 g) and 15 male wild-type mice (WT, C57BL/6 J, 22 to 23 g) aged 6 weeks were used in this study. The traffic light breeding strategy was used to obtain heterozygous mice. All mice were housed in temperature- and humidity-controlled cages with a 12:12 h light: dark cycle and had free access to food and water. They were fed a soft diet to minimize any discomfort or displacement of the orthodontic appliance. Mouse genotyping protocol was performed by polymerase chain reaction (PCR) to identify CB2^−/−^ and WT mice from the offspring of heterozygous mice. The PCR protocol was provided by Gempharmatech Co., Ltd [16, 17]. The mice were randomly allocated into two groups (Table 1)-WT orthodontic movement groups (WT OTM):0,7 and 14 days; CB2^−/−^ orthodontic movement groups (CB2^−/−^ OTM): 0, 7 and 14 days.

**Table 1:** Distribution of samples for each group

OTM (days)	WT (mice)	CB2(mice)
0	5^a^	5^a^
7	5	5
14	5	5

^a^Without force application

### OTM model

**Fig. 1:**
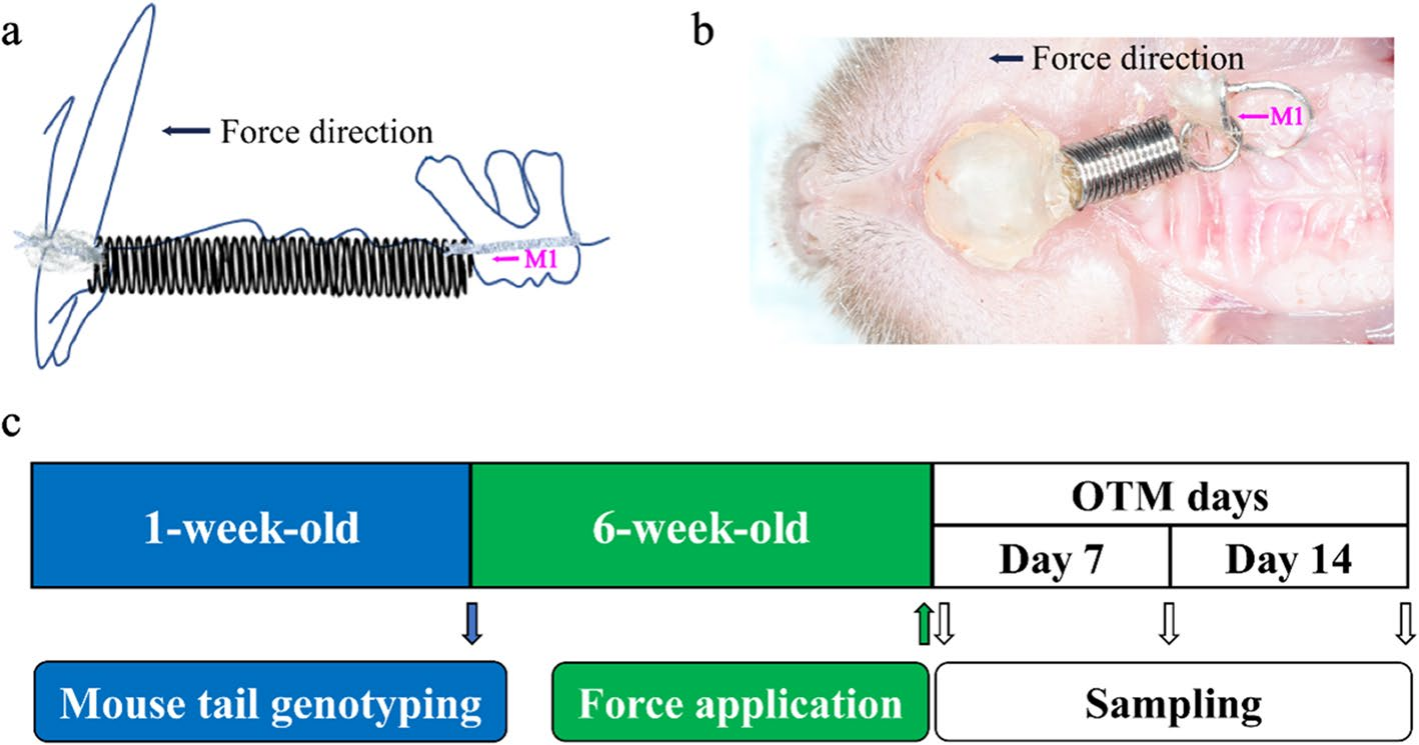
Details of the appliance resulting in OTM. **a** Conceptual diagram of the OTM model. **b** This activated nickel-titanium coil spring was ligated to maxillary first molar and incisors to provide a continuous force to induce tooth movement. **c** Flowchart of the experiment

Sodium pentobarbital (150–200 mg/kg,) was administered intraperitoneally to anesthetize the mice. Orthodontic appliances consisting of an activated nickel-titanium coil spring (MX-1109–01, Mingxing Spring Co., LTD, Chengdu) were ligated from the maxillary first molar (M1) to the central incisor to deliver a force (20N) for tooth movement (Fig. 1a). One end of the orthodontic nickel-titanium coil spring was fixed on the first molar through an orthodontic ligature wire, while the other end of the ligature wire was directly fixed on the incisor and bonded with a flowable composite resin (3 M ESPE) under anesthesia (Fig. 1b) [18–20]. The orthodontic force was applied to the mice immediately after the appliance placement. The integrity of the coil spring was examined daily to prevent any loosening or damage of the device. The degree of tooth movement was quantified and compared among all the groups (Fig. 1c).

### Tissue preparation

Mice were individually cervical dislocation for euthanasia on 0, 7 and 14 days after the procedure. The maxilla was isolated and promptly fixed in 4% paraformaldehyde solution for 24 h, and then subjected to micro-CT analysis. The samples were decalcified in 10% EDTA (pH 7.2) for at least 30 days and embedded in paraffin. Paraffin sections with a thickness of 4 μm were oriented perpendicular to the occlusal plane of the molars. The sections were stained with hematoxylin and eosin, and immunohistochemical stained for tartrate-resistant acid phosphatase.

### Tooth movement and alveolar bone quality analysis

Micro-computed tomography (SkyScan 1276, Bruker, Germany) scanning was used to obtain three‐dimensional reconstructions of the maxilla. Samples were conducted in 4% paraformaldehyde. Analyses of the bone micro architecture were carried out in a region of interest (ROI), which was interradicular area of the right maxillary first molar [21]. The alveolar bone quality indices include the bone volume per tissue volume (BV/TV), trabecular number (Tb.N), trabecular thickness (Tb.Th), and bone mineral density (BMD). The scan condition including an X-ray tube potential of 85 kV, an X-ray in tensity of 200 µA, and an exposure time of 384 ms. Reconstruction was accomplished by NRecon (version 1.7.4.2). 3D images were obtained from contoured 2D images by methods based on distance transformation of the grayscale original images (CTvox; version 3.3.0). 3D and 2D analysis were performed using software CT Analyser (version 1.18.8.0). We reconstructed sections of the center of the distal buccal root of the maxillary first molar (M1) and the mesial buccal root of the second molar (M2), and then measured the distance between the most convex points of the crowns on the sections as the tooth movement distance [22, 23].

### Tartrate Resistant Acid Phosphatase (TRAP) staining and image analysis

TRAP kit (CR2107063, Sevicebio, Wuhan) was used to analyze osteoclasts according to the manufacturer’s protocol. For quantification of the number of osteoclasts, the region of interest was chosen from the Cemento-enamel junction to the apex of the distal buccal root compression side of the first molar. Based on a previous study by Liu et al., TRAP-positive cells were quantified using Image J [24].

### Immunohistochemistry (IHC)

Deparaffinized sections were incubated with 0.3% hydrogen peroxide (H_2_O_2_) for 10 min at room temperature (RT). Enzymatic antigen retrieval was done with 0.4% pepsin (1403GR025, BioFroxx Germany) dilution for 20 min at RT. Then, they were incubated with the primary antibodies (RANKL: A13567, Abclonal Wuhan; OPG: Huaan Biological Hangzhou) overnight at 4℃. Then, the sections were incubated with appropriate secondary antibodies (PV-9001, Beijing Zhongshan Jinqiao; PV-6001 Beijing Zhongshan Jinqiao) for 0.5 h at RT. After rinsed in phosphate buffered saline (PBS), the sections were developed with the DAB (ZLI-9017, Zhongshan Golden Bridge, Beijing) and counterstained with hematoxylin. Average optical density (AOD) of immunohistochemical staining was semi-quantitatively analyzed using Image J [25].

### Statistical analysis

Statistical analysis was carried out using GraphPad Prism (GraphPad Software, INC, version 9.0 for Windows, San Diego, CA, USA). Data were presented in mean ± standard deviation. A two-sided t test was used for statistical testing of independent samples. ANOVA with Bonferroni post hoc test was used for comparison between groups. The level for statistical significance was set as *P* < 0.05. In the figures, the asterisks indicated statistically significant. *P < 0.05, ** *P* < 0.01, *** *P* < 0.001, ****and *P* < 0.0001.

## Results

### The growth and development of CB2^−/−^mice

During the 14-day experimental period, no mice died or had a loosening of the appliance. There were no significant differences in size and body weight between littermate 6-week-old CB2^−/−^ and WT mice (additional files 1: Fig. S1a-e). To confirm the working distance of the orthodontic tension device, we also measured the distance from the first permanent molar to the incisor. There was no difference in the distance between CB2^−/−^ and WT mice in 0-day groups.

### The orthodontic alveolar bone of CB2^−/−^mice

**Fig. 2:**
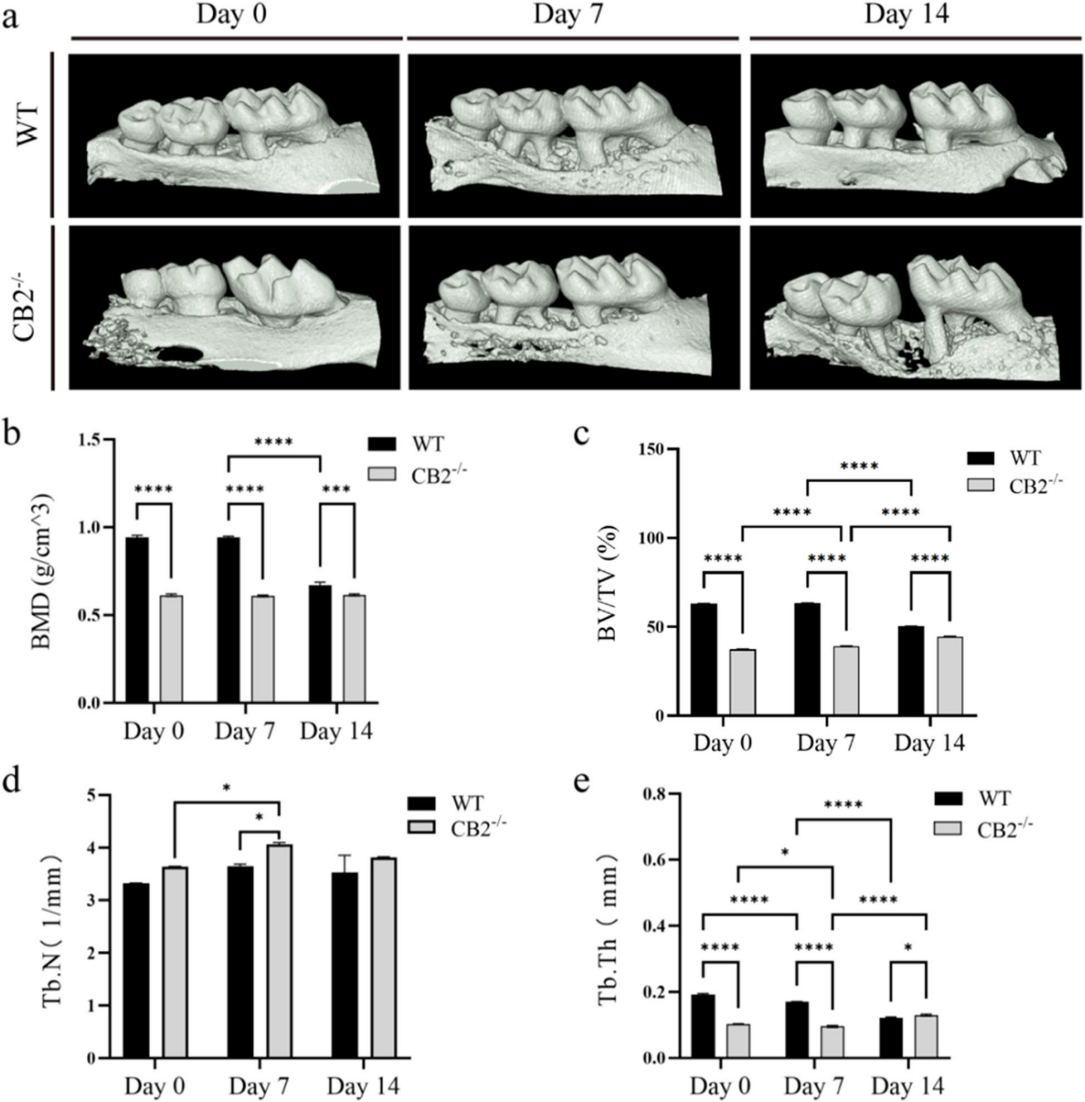
Micro-CT analysis of alveolar bone quality in CB2^−/−^ and WT mice in the maxilla. **a** Bone mineral density (BMD). **b** Statistic evaluation of Bone mineral density (BMD). **c** Statistic evaluation of Relative bone volume fraction (BV/TV). **d** Statistic evaluation of Trabecular number (Tb.N). **e** Statistic evaluation of Trabecular bone thickness (Tb.Th). Values are means ± SD. * P < 0.05, *** *P* < 0.001, and **** *P* < 0.0001

To investigate how CB2 deficiency affects orthodontic alveolar bone, we used micro-CT to quantify the bone structure and quality in CB2^−/−^ mice and WT mice (Fig. 2a). The micro-CT results showed there was significantly more alveolar bone loss in CB2^−/−^ mice than WT mice, both in the orthodontic and non-orthodontic regions (Fig. 2b). Moreover, the BMD of alveolar bone in CB2^−/−^ mice did not change in orthodontic and non-orthodontic groups (Fig. 2b). However, the BV/TV of alveolar bone increased on day 7 and reached the peak on day 14 in CB2^−/−^ mice after OTM (Fig. 2c). Likewise, although the Tb.Th decreased and the Tb.N increased on day 7, the alveolar bone quality improved gradually in CB2^−/−^ mice after OTM (Fig. 2d,e). These findings suggest that, CB2^−/−^ mice did not exhibit further bone loss with orthodontic force application within 14 days, unlike WT mice.

### The increased tooth movement in CB2^−/−^ mice

**Fig. 3:**
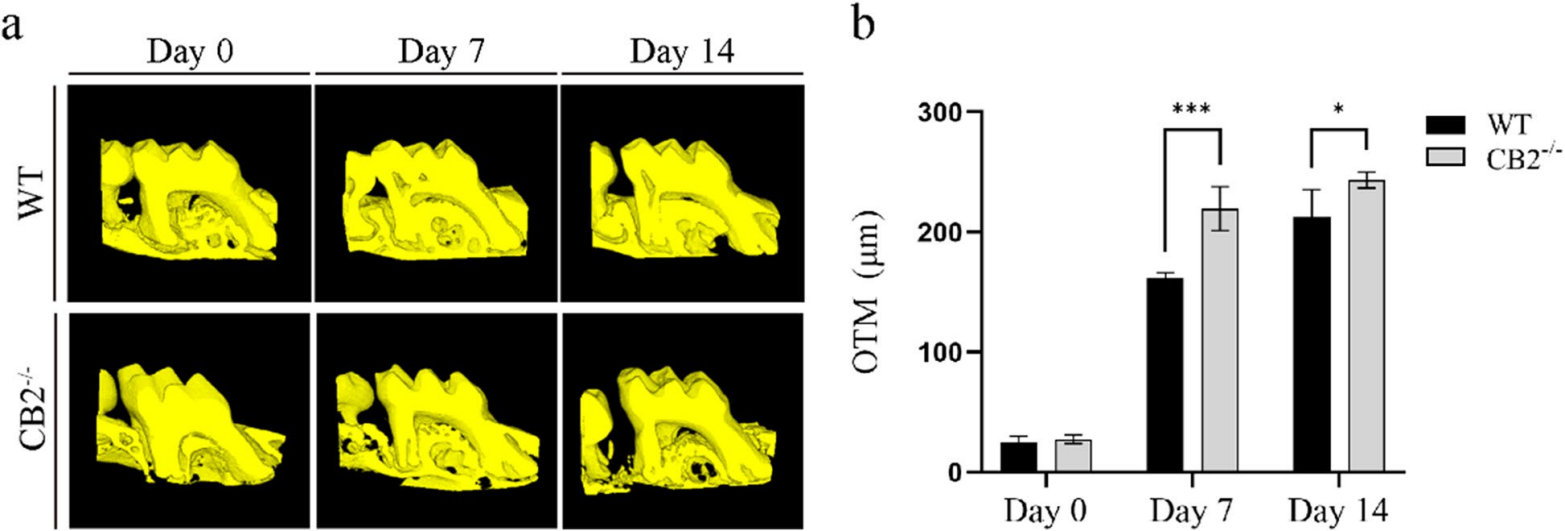
Analysis of tooth movement distance from micro-CT. **a** Reconstructed sections of the maxillary first molar and the mesial buccal root of the second molar. **b** Quantification of the OTM distance between M1 and M2 after force applying in CB2.^−/−^ and WT mice. Values are means ± SD.*P < 0.05 and *** *P* < 0.001

We analyzed the amount of OTM in CB2^−/−^ and WT mice on micro-CT cross-sections. We found obvious mesial movement of the maxillary first molar in all groups after 14 days of OTM (Fig. 3a). CB2^−/−^ mice showed a greater distance between the maxillary first and second molars than WT mice (Fig. 3b). The tooth movement distance showed an increasing trend proportional to time during application loading (Fig. 3b). Taken together, these results indicate a faster tooth movement in CB2^−/−^ mice than in WT mice.

### The increased number of osteoclasts in the orthodontic force-induced alveolar bone remodeling of CB2^−/−^ mice

#### (1) The increased number of osteoclasts in CB2^−/−^ OTM mice

**Fig. 4:**
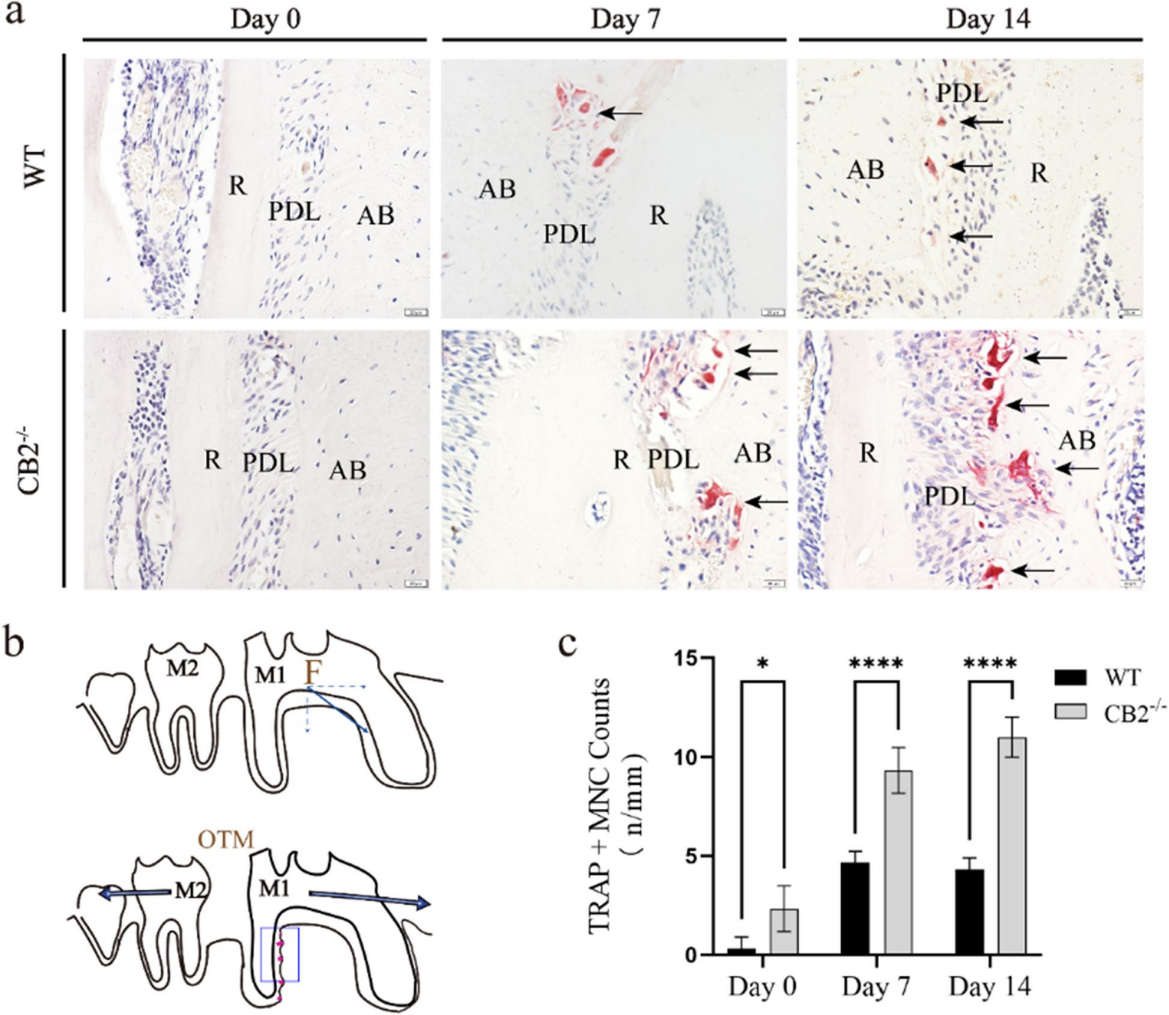
Quantitative analysis of the number of osteoclasts in the M1 pressure area after OTM in CB2^−/−^ and WT mice. **a** TRAP staining results in the distal root compression side of the first molar. Scale bar: 50 µm. R, root; PDL, periodontal ligament; AB, alveolar; ↑ (Black arrow), TRAP positive cells. **b** Illustration of the tooth movement and osteoclast distribution in the maxillary first molar after OTM. F: The direction of Orthodontic force. **c** Statistic evaluation of TRAP positive osteoclasts in ROI, data are shown as mean ± standard deviation. Values are means ± SD. * *P* < 0.05 and **** *P* < 0.0001

To examine whether CB2 was involved in the orthodontic force-induced alveolar bone resorption, TRAP staining was performed to detect the osteoclasts. The number of osteoclasts significantly increased, reaching a peak on day 14 in the alveolar bone of CB2^−/−^ mice subjected to orthodontic force, indicating that CB2 knockout enhanced the process of bone resorption during OTM by increasing the number of osteoclasts (Fig. 4).

#### (2) Increased RANKL expression and decreased OPG expression in CB2^−/−^ mice

**Fig. 5:**
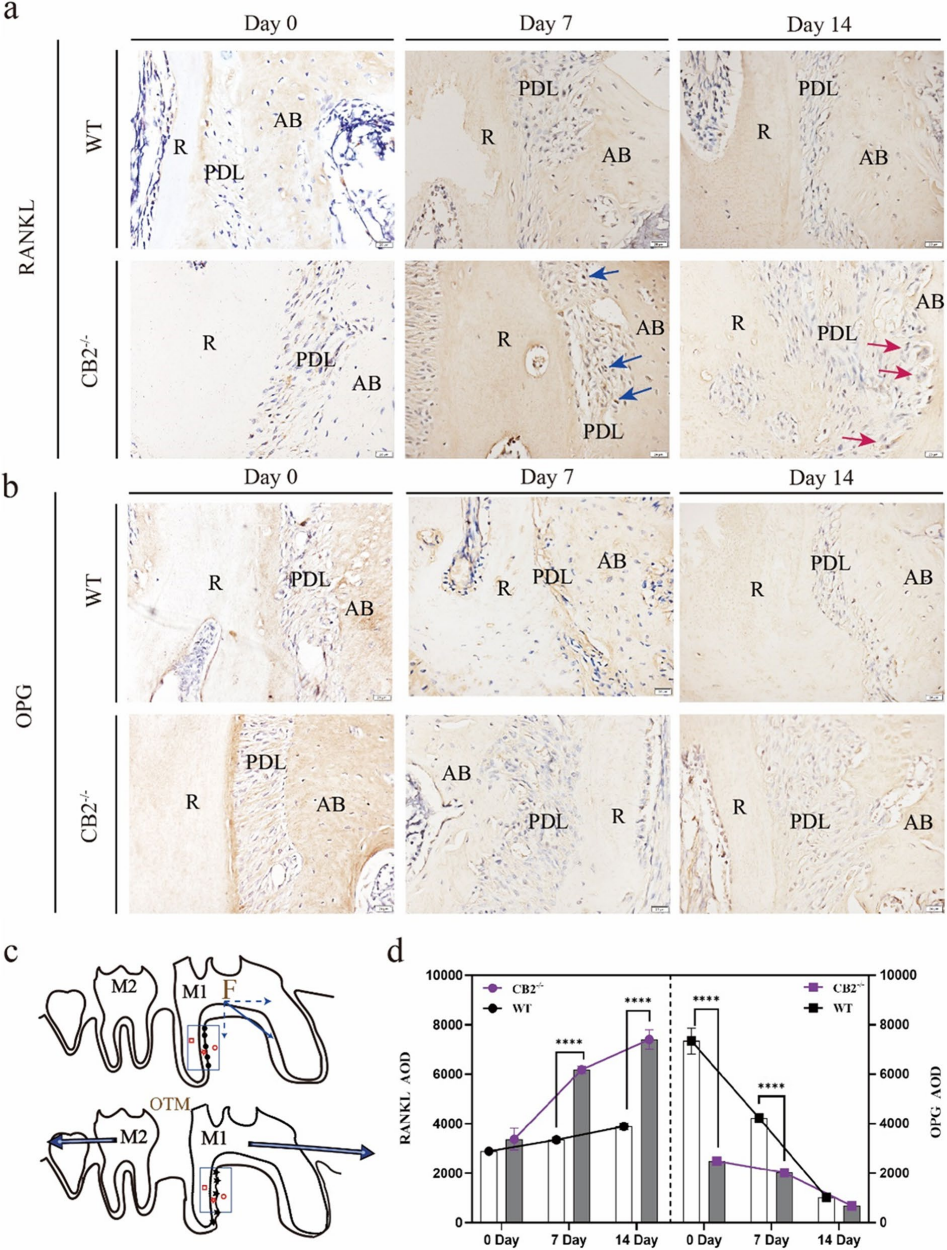
Quantitative analysis of immunohistochemistry of RANKL and OPG in the M1 pressure area after OTM in CB2.^−/−^ and WT mice. **a**, **b** IHC of OPG /RANKL staining results in the distal root compression side of the first molar mice. Scale bar: 50 µm. R, root; PDL, periodontal ligament; AB, alveolar; ↑ (Blue arrow), RANKL-positive mononuclear cells ↑ (Red arrow), RANKL-positive multinuclear cells. **c** Illustration of the M1 pressure area after OTM. F: The direction of orthodontic force. **d** Statistic evaluation of the OPG /RANKL AOD in ROI, data were shown as mean ± standard deviation. Values were means ± SD.**** *P* < 0.0001

To further understand the role of CB2 in the orthodontic force-induced alveolar bone remodeling at the molecular level, the AOD values of OPG and RANKL immunohistochemical staining were analyzed in the pressure area from parasagittal sections of the mesiobuccal and distobuccal roots of the first molars. We found that RANKL-positive multinucleated cells were also positive for TRAP staining and thus confirmed to be osteoclasts (Fig. 5a). There were a vast majority of RANKL-positive mononuclear cells in the PDL, and a vast majority of RANKL-positive multinuclear cells were distributed along the bone surface. But the OPG-positive cells were very rare (Fig. 5b). The observation area of the immunohistochemical section is shown in the figure (Fig. 5c). Compared with the control group, the CB2^−/−^ group displayed a lower OPG AOD and a higher RANKL AOD (Fig. 5d). It indicated that *Cnr2* gene knockout upregulated the process of bone resorption during OTM by accelerating osteoclast differentiation.

## Discussion

This is the first study to demonstrate that bone resorption is increased in CB2^−/−^ mice during OTM. Since OTM depends on bone metabolism, and this process can be influenced by various medications [26], it is important and clinically relevant to investigate the role of CB2 in OTM.

To investigate the effects of CB2, we used CB2^−/−^ knock-out mice with a C57 BL/6 J background as a model of the *Cnr2* gene deletion. We induced tooth movement, which is a reliable way of studying the compression and tension roles in periodontal remodeling, resulting in well-described changes in alveolar bone quality, osteoclast/osteoblast regulation, and bone formation/resorption. When the intensity of orthodontic force reaches a certain threshold, the number of osteoclasts reaches its maximum, and higher force levels do not enhance tooth movement or increase root resorption [27]. Therefore, to achieve optimal tooth movement and osteoclast activation, a consistent force value of 20 g was applied for all force devices. Since bone resorption on the pressure side has been reported in the late stage of OTM [28], we selected a time period of 2–4 weeks after the force application. The duration was crucial for studying the processes of bone modelling during OTM; therefore, we set different time points.

Micro-CT analysis was used to compare tooth movement distance and alveolar bone resorption among groups at different time points. Teeth usually moved the fastest in the first 3 weeks, and even without reactivating the orthodontic spring coil, tooth movement could continue at a lower rate, indicating that bone remodeling persisted if the force was maintained [29]. In this study, the faster tooth movement was found in CB2^−/−^ OTM groups than the control groups. This difference was most evident on the 7th day of tooth movement, and lasted until the 14th day. We found that CB2 deficiency did not affect the tooth size or position in mice, as there was no difference in the distance between the maxillary first molar to the second molar in WT and CB2 − / − mice before tooth movement. This showed that CB2 played a role in bone remodeling after the force applied. These results suggest that CB2 deletion led to accelerated tooth movement, and this significant change occurred gradually with the application of force. In our study, we detected more alveolar bone resorption in CB2^−/−^ mice than in WT mice (Fig. 3). We suggested that our findings were related to the effect of *Cnr2* knockout on the bone resorptive process. Because bone metabolism involves both osteoblast and osteoclast activity, it is possible that *Cnr2* knockout may affect other bones throughout the body. CB2 was a regulator of bone remodeling partly by enhancing bone formation through upregulating osteoblast proliferation and activity and partly by reducing bone resorption through decreasing osteoclast differentiation and activity. Previous studies showed that CB2 had two effects on osteoclast/osteoblast and alveolar bone structures: CB2 deletion caused significant periodontal bone loss and osteoclast formation from bone marrow macrophages (BMCs), which was reversed by CB2 antagonists. In CB2^−/−^ mice, loss of function also led to osteoporosis in aged CB2^−/−^ mice [1, 6, 9, 10, 30].

We found that the speed of OTM was mainly influenced by alveolar bone quality. In this study, quantification of BMD revealed that significant reductions of BMD could accelerated OTM in CB2^−/−^ mice. In fact, given that the alveolar bone remodeling was dominated by bone resorption, the BMD is decreased even in the WT group after 14-day of appliance [31]. Alveolar bone loss was inevitable after OTM. Consistent with our study in WT mice, bone histological and histomorphometry analyses in previous studies have shown that alveolar bone volume was lower in appliance groups than in their control groups [32–34]. Interestingly, we observed that BMD values remained stable in both orthodontic and non-orthodontic alveolar bone in CB2^−/−^ mice. The possible explanation was that alveolar bone remodeling is more efficient in mice lacking CB2, but also more prone to cause alveolar bone deterioration [35]. Therefore, the application of tooth movement forces may not have a significant impact on alveolar bone in this condition, since the alveolar bone is already undergoing rapid remodeling. Indeed, differences in alveolar bone volume depend on the balance between bone formation and bone resorption. Normally, when a force is applied to a tooth, periodontium expansion and bone formation occur on the tension side and alveolar bone resorption occurs on the compression side. These responses were observed in control but not in the CB2^−/−^ mice. Taken together, we conducted that CB2 was involved in bone modeling during OTM in the animal model.

During OTM, the mechanical force induced inflammatory cytokine release and osteoclast recruitment to resorb bone on the compression side. CB2 deletion altered the inflammatory signaling and increased proinflammatory cytokine release, which may account for the increased bone resorption in CB2^−/−^ mice vs WT mice in histologic findings. Research has found that periodontal osteoclast differentiation is related to the ratio of RANKL/ OPG [36]. Klein et al. reported that dronabinol, which can combine with CB2, inhibited alveolar bone remodeling in tooth movement of rats. They found that dronabinol reduced the number of osteoclasts and osteoblasts in the pressure and tension sides of the alveolar bone, as well as the expression of RANKL, OPG [15]. Our results are consistent with their findings, as we also observed that CB2^−/−^ mice showed increased tooth movement, osteoclast number, RANKL-positive cells and decreased OPG-positive cells compared to WT mice during OTM. To investigate the role of CB2 in alveolar bone remodeling induced by orthodontic force, we further quantized TRAP + osteoclasts in both CB2^−/−^ and WT mice to study the bone resorption process. The number of osteoclasts increased significantly in both appliance groups compared to control groups during 21 days of OTM. A previous study reported similar results, where all animal appliance groups showed increased number of osteoclasts [32]. The genomic analysis of Raw 264.7 revealed that *Cnr2* was negatively correlated to osteoclasts [37, 38]. However, research showed that there was no significant difference in the number of TRAP positive osteoclasts in alveolar bone between CB2^−/−^ and WT [30]. OPG and RANKL have opposite effects by competitively binding to rank receptors on the osteoclast membrane. RANKL was upregulated during orthodontic bone remodeling, with high expression on osteoclasts, but the research on the expression pattern of OPG was insufficient. Therefore, CB2 negatively regulated osteoclast differentiation in orthodontic alveolar bone remodeling.

The regulatory network of orthodontic tooth movement is poorly understood, and most clinical orthodontic patients are still healthy young patients. Further exploring the role of CB2 in orthodontic alveolar bone remodeling will provide a new insight for clinical applications such as accelerating tooth movement to shorten the treatment duration, or treating orthodontic patients with bone abnormalities, etc. In the present study, we found that CB2 plays an important role in tooth movement and alveolar bone remodeling, and that pharmacological interventions targeting CB2 receptors may help enhance these processes. There are currently some pharmacological possibilities to target CB2, such as JWH133 is a synthetic CB2-selective agonist that has polypharmacological properties and therapeutic potential [39]. However, the clinical use of drugs targeting CB2 receptors also requires consideration of their pros and cons, ethical and legal issues.

## Conclusion

OTM is a unique alveolar bone remodeling process induced by mechanical force stimulation, which is characterized by bone resorption on the pressure side and bone formation on the tension side. Accelerating OTM is an important goal in clinical orthodontics, as it can shorten treatment time and reduce complications. Currently the use of cannabis has become more popular, but the role of cannabis on CB2 receptors and tooth movement and alveolar bone remodeling is unclear and requires further investigation. This study found that CB2^−/−^ mice had faster OTM and bone remodeling than WT mice. CB2 is a key regulator of osteoclast recruitment and activation, which play crucial roles in bone metabolism. These results suggest that the absence of CB2 may accelerate OTM and alveolar bone remodeling by modulating RANKL/ OPG and osteoclastogenesis. Therefore, targeting CB2 may be a novel strategy for enhancing OTM and alveolar bone remodeling in clinic. However, more studies are needed to elucidate the detailed molecular pathways involved in CB2-mediated regulation of OTM and alveolar bone remodeling, as well as the potential side effects of CB2 inhibition on other physiological processes.

### Supplementary Information

**Additional file 1:** **Fig. S1.** The growth and development of CB2^-/-^ mice. a PCR gel electrophoresis. b, c Body weight and body length of 6-week-old wild-type and CB2^−/−^ littermates. d, e There were no significant differences in body weights and length between two genotypes at 6 weeks. Values are means ± SD. NS, not significant. Full-length blots/gel is presented in Fig. S3. **Fig. S2.** Micro-CT analysis of 5-month-old mouse femur. a A WT mouse, B CB1^−/−^ mouse, C CB2^−/−^ mouse. b Statistic evaluation of Bone mineral density (BMD), Relative bone volume fraction (BV/TV) and trabecular number (Tb.N). Values are means ± SD. * *P* < 0.05, *** *P* < 0.001, and **** *P* < 0.0001. **Fig. S3.** Full-length PCR gel electrophoresis.

## Data Availability

All data generated or analyzed during this study are available from corresponding authors to any reader directly upon reasonable request.

## Abbreviations

CB2: Cannabinoid receptor 2
WT: Wild type
OTM: Orthodontic tooth movement
TRAP: Tartrate-resistant acid phosphatase
RANKL: Receptor activator of NF-κB ligand
OPG: Osteoprotegerin
PCR: Polymerase chain reaction
M1: Maxillary first molar
M2: Maxillary second molar
BV/TV: Bone volume per tissue volume
Tb.N: Trabecular number
Tb.Th: Trabecular thickness
BMD: Bone mineral density
IHC: Immunohistochemistry
RT: Room temperature
H_2_O_2_: Hydrogen peroxide
AOD: Average optical density
PBS: Phosphate buffered saline
BMCs: Bone marrow macrophages
