# Supplementary material for: The role of cannabinoid receptor 2 in bone remodeling during orthodontic tooth movement
Source: BMC Oral Health. 2024 Jan 4;24:23. doi: 10.1186/s12903-023-03810-5 (PMC10768142; doi:10.1186/s12903-023-03810-5)
Supplement: Supplementary file 1 — Additional file 1: Fig. S1. The growth and development of CB2-/- mice. a PCR gel electrophoresis. b, c Body weight and body length of 6-week-old wild-type and CB2−/− littermates. d, e There were no significant differences in body weights and length between two genotypes at 6 weeks. Values are means ± SD. NS, not significant. Full-length blots/gel is presented in Fig. S3. Fig. S2. Micro-CT analysis of 5-month-old mouse femur. a A WT mouse, B CB1−/− mouse, C CB2−/− mouse. b Statistic evaluation of Bone mineral density (BMD), Relative bone volume fraction (BV/TV) and trabecular number (Tb.N). Values are means ± SD. * P < 0.05, *** P < 0.001, and **** P < 0.0001. Fig. S3. Full-length PCR gel electrophoresis. [file 12903_2023_3810_MOESM1_ESM.docx]

**Additional files 1:**


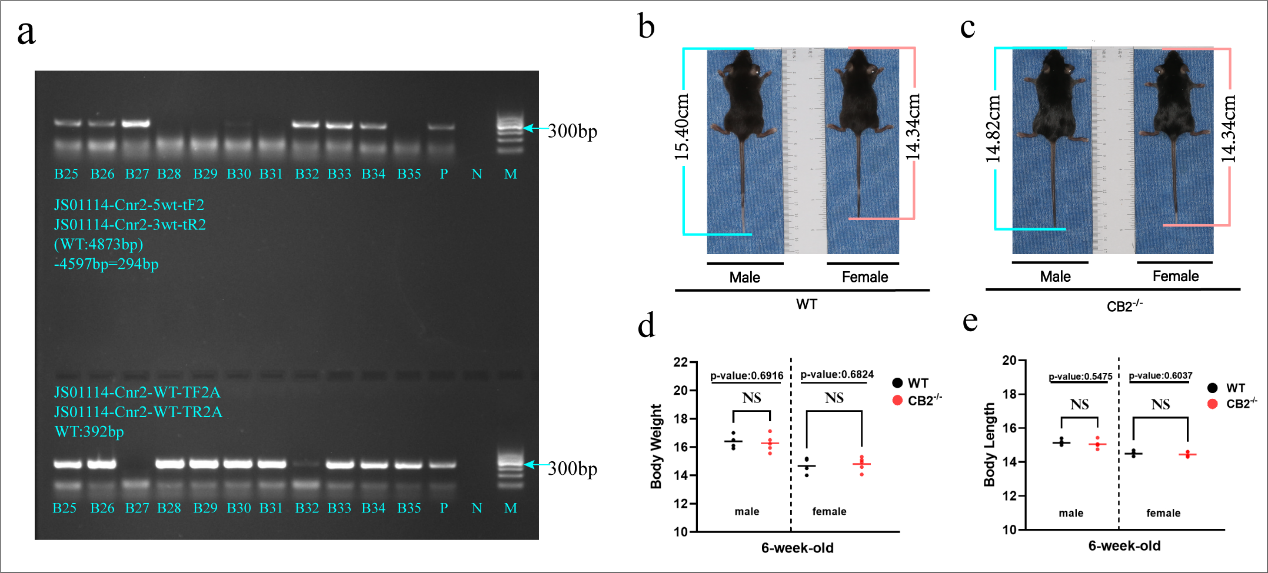
**Fig. S1** The growth and development of CB2^-/-^ mice. a PCR gel electrophoresis. b, c Body weight and body length of 6-week-old wild-type and CB2^−/−^ littermates. d, e There were no significant differences in body weights and length between two genotypes at 6 weeks. Values are means ± SD. NS, not significant. Full-length blots/gel is presented in Fig. S3.

The micro-CT results of mouse femur showed that (Fig. S2), compared with WT mice, CB1^−/−^ and CB2^−/−^ mice had significantly lower bone density and bone mass, and CB2^−/−^ mice had more pronounced reduction, indicating that CB2 had more prominent effect on bone mass maintenance.


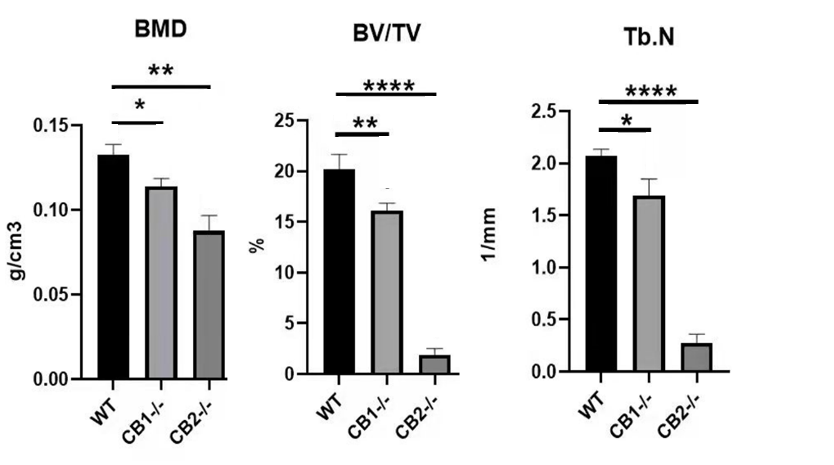

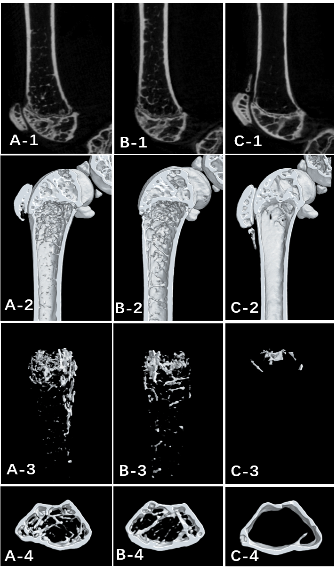


**a**

**b**

**Fig. S2** Micro-CT analysis of 5-month-old mouse femur. a A WT mouse, B CB1^−/−^ mouse, C CB2^−/−^ mouse. b Statistic evaluation of Bone mineral density (BMD), Relative bone volume fraction (BV/TV) and trabecular number (Tb.N). Values are means ± SD. * P < 0.05, *** P < 0.001, and **** P < 0.0001.


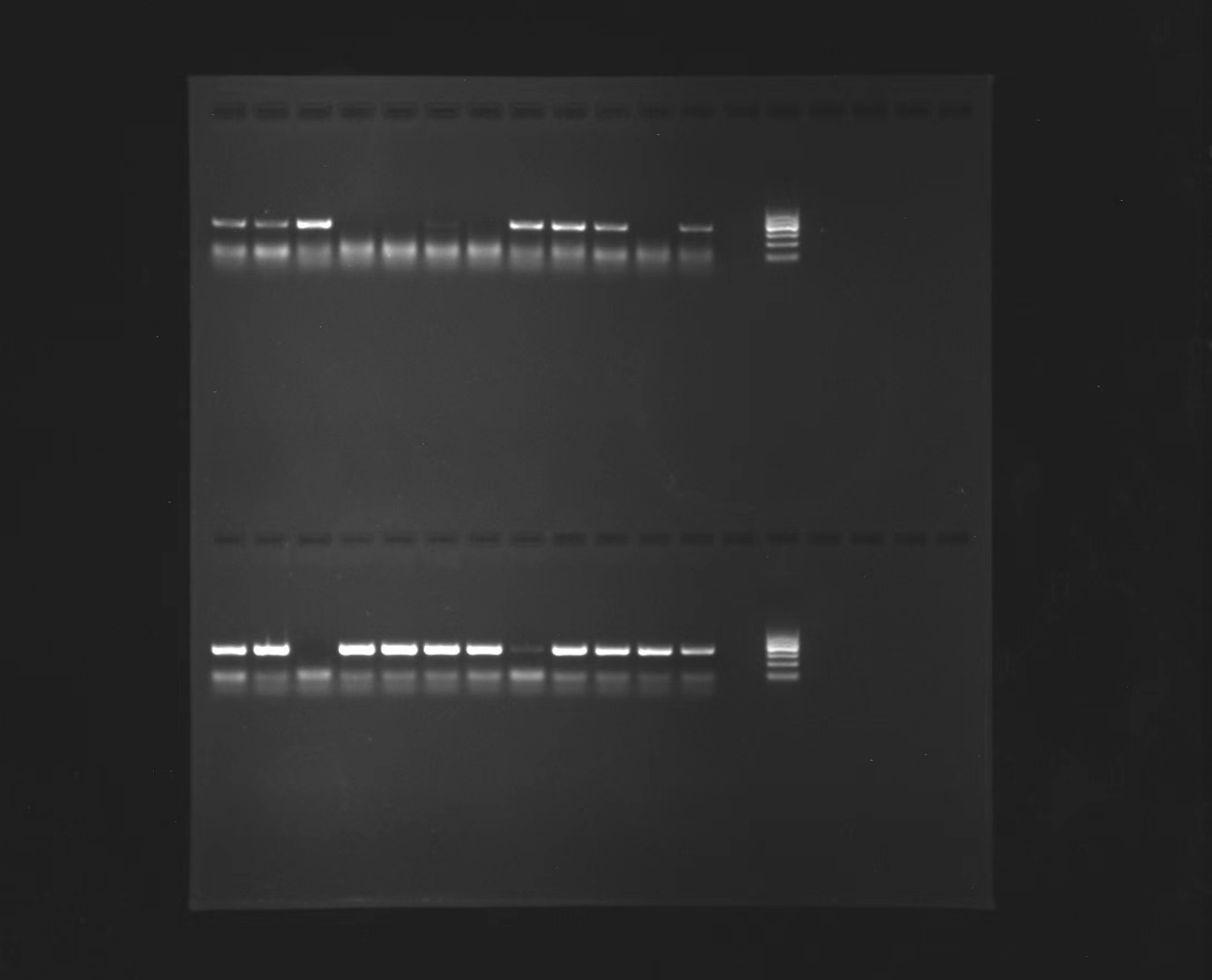


**Fig. S3** Full-length PCR gel electrophoresis.
